# The Treatment of Suppuration by Bicarbonate of Soda

**Published:** 1899-01

**Authors:** 


### THE TREATMENT OF SUPPURATION BY BICARBONATE OF SODA.

Brucker (*Thèse de Bordeaux*) has made a study of a fact observed by himself,—namely, the influence of the reaction of the blood in the healing of certain conditions. Bearing in mind that the normal alkalinity of the blood shows important variations according to sex, age, and as to whether the blood is arterial or venous in origin, and the diet to which the patient has been addicted, and that in certain pathological conditions these variations are very marked, so that a reduction in the normal alkalinity is observed in certain cases of febrile reaction due to bacterial intoxication, he has found that certain artificial intoxications can be combated by raising the alkalinity of the blood by the injection of alkaline serum. Going on these grounds, Brucker has principally investigated the influence of alkaline dressings in the treatment of local inflammatory affections, and according to his observations such a dressing, whether moist or dry, very rapidly reduces the inflammation, suppurative or otherwise, and causes rapid healing of wounds. This seems independent of any antiseptic property in the proper sense of the word. The method employed by him is to apply the dressing of absorbent wool on ordinary principles, using merely a two-per-cent. solution of bicarbonate of soda, or in some cases vaseline and bicarbonate (1 in 25), or the soda may be applied directly in the form of a powder. He finds that strong solutions do not act more quickly than a two per cent., showing that the chief agent is the alkali, and not any antiseptic principle. The same method may be applied for purulent otitis, etc.—*British Medical Journal*, June 13, 1898.

